# The *Rhododendron* Plant Genome Database (RPGD): a comprehensive online omics database for *Rhododendron*

**DOI:** 10.1186/s12864-021-07704-0

**Published:** 2021-05-22

**Authors:** Ningyawen Liu, Lu Zhang, Yanli Zhou, Mengling Tu, Zhenzhen Wu, Daping Gui, Yongpeng Ma, Jihua Wang, Chengjun Zhang

**Affiliations:** 1 Germplasm Bank of Wild Species, Kunming Institute of Botany, Chinese Academy of Sciences, Kunming, 650201 China; 2 University of Chinese Academy of Sciences, Beijing, 100049 China; 3 The Flower Research Institute, Yunnan Academy of Agricultural Sciences, Kunming, 650205 China; 4 National Engineering Research Center for Ornamental Horticulture, Kunming, 650205 China; 5 Key Laboratory of Economic Plants and Biotechnology, Yunnan Key Laboratory for Wild Plant Resources, Kunming Institute of Botany, Chinese Academy of Sciences, Kunming, 650201 China; 6 Yunnan Key Laboratory for Integrative Conservation of Plant Species with Extremely Small Populations, Kunming Institute of Botany, Chinese Academy of Sciences, Kunming, 650201 China; 7 Haiyan Engineering & Technology Center, Kunming Institute of Botany, Chinese Academy of Sciences, Jiaxing, 314300 China

**Keywords:** *Rhododendron*, Horticulture plant, Database, Functional genomics

## Abstract

**Background:**

The genus *Rhododendron* L*.* has been widely cultivated for hundreds of years around the world. Members of this genus are known for great ornamental and medicinal value. Owing to advances in sequencing technology, genomes and transcriptomes of members of the *Rhododendron* genus have been sequenced and published by various laboratories. With increasing amounts of omics data available, a centralized platform is necessary for effective storage, analysis, and integration of these large-scale datasets to ensure consistency, independence, and maintainability.

**Results:**

Here, we report our development of the *Rhododendron* Plant Genome Database (RPGD; http://bioinfor.kib.ac.cn/RPGD/), which represents the first comprehensive database of *Rhododendron* genomics information. It includes large amounts of omics data, including genome sequence assemblies for *R. delavayi*, *R. williamsianum,* and *R. simsii*, gene expression profiles derived from public RNA-Seq data, functional annotations, gene families, transcription factor identification, gene homology, simple sequence repeats, and chloroplast genome. Additionally, many useful tools, including BLAST, JBrowse, Orthologous Groups, Genome Synteny Browser, Flanking Sequence Finder, Expression Heatmap, and Batch Download were integrated into the platform.

**Conclusions:**

RPGD is designed to be a comprehensive and helpful platform for all *Rhododendron* researchers. Believe that RPGD will be an indispensable hub for *Rhododendron* studies.

## Background

*Rhododendron* L. is the largest genus in the Ericaceae, which is the largest genus of woody angiosperms in China [1]. The genus is widely distributed throughout the Northern Hemisphere from tropical Southeast Asia to northeastern Australia [2]. There are more than 1000 species of *Rhododendron* worldwide, approximately 600 of which encompassing nine subgenera are found in China [3, 4]. Southwestern China and the eastern Himalayas are considered as centers of *Rhododendron* diversification and differentiation [5]. Rhododendrons are considered to have great ornamental and medicinal value [6, 7].

Horticultural interest in *Rhododendron* can be traced back at least several centuries, owing in part to their bright coloring and elegant posture [8, 9]. In China, its introduction and cultivation was first documented in poetry from the Tang dynasty, and rhododendrons have long been developed as one of the ten national-traditional ornamental flowers [8]. The breeding history began with gardening enthusiasts in Western countries in the late eighteenth century [9]. Currently, there are over 28,000 cultivars of *Rhododendron* [10], which are widely cultivated in many regions such as Asia, America, and Europe [6]. Most wild rhododendrons are found in regions with temperate climates, high rainfall, humid atmosphere, and organic acid soils with low nutrient composition [11]. Furthermore, most varieties are derived through crossbreeding by gardening enthusiasts according to their preference for ornamental traits. In general, breeding goals have previously been focused mostly on ornamental characteristics rather than adaptability and resistance, resulting in a disconnect between existing varieties and market demands. Therefore, a challenge for *Rhododendron* breeding is the development of varieties capable of adapting to environments with cold winters, hot summers, lower rainfall and humidity, and less optimal soils [12].

Additionally, the genus *Rhododendron* has a long history in traditional medicine [7]. Phytochemists have demonstrated interest in *Rhododendron* species due to their abundance of secondary metabolites [13]. Currently, approximately 200 compounds, mostly flavonoids and diterpenoids, have been isolated from *Rhododendron*. Some of the isolates have demonstrated intriguing bioactivity [14, 15]. For example, diterpenoids isolated from the flowers, roots, and fruits of *R. molle* exhibit significant anticancer, antiviral, antinociceptive, immunomodulatory, and sodium channel antagonistic activities.

With the rapid development of sequencing and genomic editing technology, molecular design breeding has become a more efficient and accurate plant breeding method [16]. Elucidation of the genetic mechanisms associated with ornamental traits (flower color, flower shape, etc.), adaptability, resistance, secondary metabolism, etc. will be a helpful and necessary foundation for more practical *Rhododendron* breeding. A great deal of omics data concerning *Rhododendron* have been accumulated to date and several rhododendron genomes have been sequenced. The *R. delavayi* genome sequence was released in 2017 [17], *R. williamsianum* in 2019 [18], and *R. simsii* in 2020 [19]. In addition, relevant transcriptomic data have also been published in recent years [20–24]. Progress in the development of high-throughput sequencing technology has greatly accelerated studies on *Rhododendron* [17–24]. These large genomic data sets provide a new perspective for understanding biological traits such as ornamentation, adaptability, resistance, and secondary metabolism for breeders and phytochemists alike.

*Rhododendron* omics data sets are currently distributed in public databases that are easily accessible [25, 26]. However, processing these data is a considerable challenge for research groups with limited bioinformatics experience. To address this problem, we have constructed a comprehensive database for data storage, categorization, online analysis, and visualization of *Rhododendron* omics data sets.

Here, we present the *Rhododendron* Plant Genome Database (RPGD; http://bioinfor.kib.ac.cn/RPGD/), a data center for *Rhododendron* functional genomics researchers. The database integrates the three released genome sequences, expression profiles, functional annotations, gene family ontologies, simple sequence repeats, chloroplast genome assemblies, and gene homology information. We have also incorporated bioinformatics tools such as BLAST, JBrowse, Flanking Sequence Finder, Genome Synteny Browser, Ortholog Gene Finder, Expression Heatmap, and Batch Download into the user interface. The interface is designed to be simple and user-friendly. We suggest that RPGD will be of great convenience as a “one-stop shop” to a wide range of *Rhododendron* researchers.

## Construction and content

### Genomic data

Currently, three reference genome sequences of *Rhododendron* - *R. delavayi*, *R. williamsianum* and *R. simsii* - are hosted in RPGD (Table 1). The genome sizes are 695 Mb, 532 Mb and 529 Mb, respectively; and the scaffold N50 are 637.83 kb, 218.8 kb and 36.3 Mb, respectively [17–19]. The genome of *R. simsii* was sequenced by PacBio long-read sequencing technology [19], while *R. delavayi* and *R. williamsianum* were based on next-generation sequencing [17, 18]. We downloaded the genome assembly, general feature format (GFF3), coding sequence (CDS), and protein sequence (PEP) of *R. delavayi* (http://gigadb.org/dataset/100331) from the GigaScience database [17, 26], and for *R. williamsianum* (https://www.ncbi.nlm.nih.gov/assembly/GCA_009746105.1) and *R. simsii* (https://www.ncbi.nlm.nih.gov/assembly/GCA_014282245.1) from NCBI [18, 19, 25].

**Table 1:** Data statistics in RPGD database

Data type	Number
**Gene**	
Genes for *R. delavayi*	32,938
Genes for *R. williamsianum*	23,559
Genes for *R. simsii*	32,999
**Genome**	
Scaffolds for *R. delavayi*	193,091
Chromosomes for *R. williamsianum*	13
Chromosomes for *R. simsii*	13
**Gene ontology (GO)**	
*R. delavayi*	
Genes	21,361
Annotations	805,276
*R. williamsianum*	
Gene	17,658
Annotations	687,600
*R. simsii*	
Genes	22,235
Annotations	785,704
**Gene Family**	
Gene families for *R. delavayi*	4168
Gene families for *R. williamsianum*	3546
Gene families for *R. simsii*	3742
**Transcription factor (TF) and Transcriptional regulators (TRs)**	
TFs and TRs for *R. delavayi*	2104
TFs and TRs for *R. williamsianum*	1622
TFs and TRs for *R. simsii*	2156
**Simple sequence repeat (SSR)**	
SSRs for *R. delavayi*	361,268
SSRs for *R. williamsianum*	230,013
SSRs for *R. simsii*	358,705
**Chloroplast genome assemblies**	
*R. delavayi* chloroplast genome assembly	2
*R. pulchrum* chloroplast genome assembly	1
**InterPro**	
Annotated to InterPro for *R. delavayi*	77,221
Annotated to InterPro for *R. williamsianum*	60,834
Annotated to InterPro for *R. simsii*	81,654
**Gene expression**	
RNA-Seq for *R. delavayi*	2
**Genomic synteny**	2913
**OrthoFinder orthologous/paralogs group**	18,048

### Transcriptomic data

All publicly available RNA-Seq datasets in the NCBI Sequence Read Archive (SRA) database, including data from two projects and 19 samples, were obtained. One transcriptomics project was related to drought stress (4 samples) while the other was related to the flower bud in different dormancy statuses (15 samples) [23] (Table 1). Both projects focused on *R. delavayi*.

We processed and analyzed the RNA-Seq datasets by a standard pipeline method. First, we used the SRA Toolkit [27] to convert the data format to FASTQ and low-quality reads were removed from raw reads by Trimmomatic [28]. We then employed Tophat2 [29] to map all clean reads onto the reference genome (*R. delavayi*) with default parameters, which were assembled using Cufflinks (version 2.2.1) using the reference genome as a guide [30]. Combined transcriptome assemblies were generated using Cuffmerge. Based on the alignments, the read counts of each gene were calculated and normalized to fragments per kilobase of transcript per million mapped fragments (FPKM) values in Cuffdiff. Mean and standard errors of the FPKM values were derived for the biological replicates.

### Gene model and function annotation

A total of 89,496 protein-coding genes were collected from the downloaded data mentioned in the genomic data, including 32,938 from *R. delavayi*, 23,559 from *R. williamsianum,* and 32,999 from *R. simsii*. The protocol for annotating protein-coding genes is described as follows. Firstly, protein-coding genes were annotated using two software packages, eggNOG-mapper [31, 32] and InterProScan with default parameters [33]. Then, the results from the two different tools were combined and redundant annotations were removed to obtain complete and precise GO annotations using homemade scripts. The protein sequences were aligned against the NCBI non-redundant (nr), UniProt (Swiss-Prot and TrEMBL), and *Arabidopsis* protein (TAIR) databases using the BLASTP command of DIAMOND with an E-value cutoff of 1e^− 5^ [34]. The BLASTP results against the UniProt and TAIR databases were then fed to the AHRD program (https://github.com/groupschoof/AHRD) to obtain concise, precise, and informative gene function descriptions. All BLASTP results are shown on the detailed gene page. All of these protein sequences were further compared against the InterPro database using InterProScan to identify functional domains [33].

As a result, the genes from *R. delavayi* were functionally annotated to 805,276 on GO database and 77,221 on InterPro. The *R. williamsianum* gene were functionally annotated to 687,600 on GO and 60,834 on InterPro. The *R. simsii* genes were functionally annotated to 785,704 on GO and 81,654 on InterPro (Table 1).

**Fig. 1:**
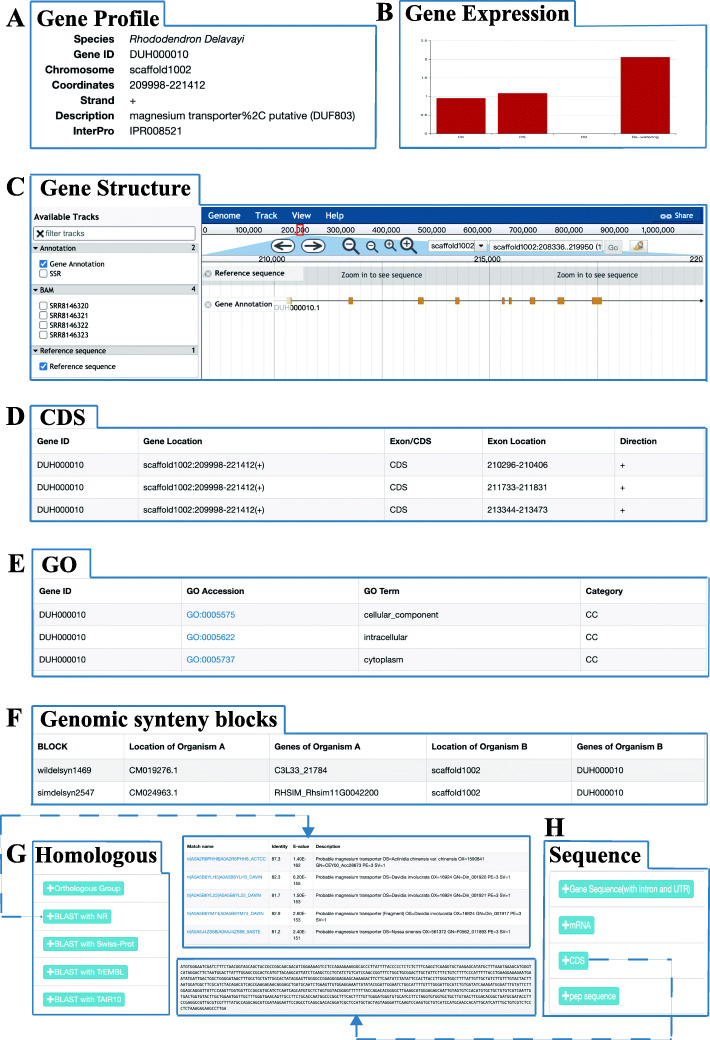
Gene feature page in RPGD. **a** Overview of gene profile information including species, gene ID, location, description, InterPro and gene family. **b** Expression profiles. **c** JBrowse gene visualization. **d** Exon/CDS information of gene. **e** GO annotation. **f** Genomic synteny blocks. **g** Homologous genes information in 6 organisms and BLASTP results against the nr-NCBI, UniProt and TAIR databases. **h** Gene/mRNA/CDS/protein sequences

These genes were used as a “data hub” to link all data types (Fig. 1), including gene summary information (species, gene ID, location, description, InterPro and gene family) (Fig. 1a), expression profiles (Fig. 1b), JBrowse gene visualization (Fig. 1c), gene exon/CDS information (Fig. 1d), GO annotation (Fig. 1e), genomic synteny blocks (Fig. 1f), homologous genes and BLASTP results against the nr-NCBI, UniProt and TAIR databases (Fig. 1g), gene/mRNA/CDS/protein sequences (Fig. 1h). All information mentioned here is shown on an integrated interface to allow users to browse conveniently.

### Transcription factors and transcriptional regulators

The iTAK package was used to identify transcription factors (TFs) and transcriptional regulators (TRs) in the three *Rhododendron* genomes and all candidates were classified into different gene families using the default parameters [35]. Thus, *R. delavayi* contains 1662 TFs and 442 TRs, *R. williamsianum* contains 1261 TFs and 361 TRs, and *R. simsii* contains 1740 TFs and 416 TRs (Table 1).

### Orthologous/paralogs group

OrthoFinder [36, 37] was employed to identify orthologous and paralogous genes by using default parameters among *R. delavayi*, *R. williamsianum*, *R. simsii*, *Actinidia chinensis* [38], *Camellia sinensis* [39] and *Arabidopsis thaliana* [40]. In total, 18,048 orthologous groups were identified. To ensure that the inference of orthologous genes was sufficiently accurate, we extracted 985 groups of single-copy orthologs to construct the “Orthologous Groups” module (Table 1). We also used OrthoFinder to search for pairwise homologous genes between the three *Rhododendron* genomes and *A. thaliana* respectively [36, 37]*.* We considered the genes of each orthologous group as belonging to one gene family and mapped gene family information from *A. thaliana* to *R. delavayi* (4168 gene families), *R. williamsianum* (3546 gene families)*,* and *R. simsii* (3742 gene families).

### Simple sequence repeats

Simple sequence repeats (SSRs) were identified in *R. delavayi*, *R. williamsianum* and *R. simsii* by MISA with default parameters; the total number were 361,268, 230,013, and 358,705, respectively [41] (Table 1). We also used Primer3 with default parameters to design primers for SSRs and the primers can be displayed on the SSR detail page [42].

### Chloroplast genomes

We also collected full-length chloroplast genomes of *R. delavayi* and *R. pulchrum* from the NCBI database [43–45]. RPGD hosts two complete chloroplast genome assemblies of *R. delavayi*. One of them is 193,798 bp in length, and 123 genes were annotated, including 80 protein-coding genes, 35 tRNA genes, and 8 rRNA genes [43]. The other is 202,169 bp in length, a total of 137 genes were found, including 88 protein-coding genes, 41 tRNAs, and 8 rRNAs [44]. The chloroplast genome of *R. pulchrum* is 136,249 bp in length, and it contains 73 genes, comprising 42 protein-coding genes, 29 tRNA genes, and 2 rRNA genes [45] (Table 1).

### Syntenic relationships among *R. delavayi*, *R. williamsianum* and *R. simsii*

We identified syntenic blocks and homologous gene pairs in the three *Rhododendron* genomes. Protein sequences were first aligned against each other (pairwise comparisons) using BLASTP with an E-value cutoff of 1e^− 5^ [46]. Based on the BLASTP results and gene positions, syntenic blocks were determined using MCScanX with default parameters [47]. A total of 2913 syntenic blocks and 55,590 homologous genes were identified (Table 1) with detail presented in the “Tools/Genome Synteny” module. Users should note that the current assembly of draft genomes and annotations might affect the results of syntenic relationships, and we will update the data when new versions become available.

### Implementation

RPGD was constructed using the LAMP framework, including Apache2 (a free and open-source cross-platform web server software; https://www.apache.org/), MariaDB (a relational database management system; https://mariadb.org/), and PHP (a popular general-purpose scripting language; https://www.php.net/). All data were stored on a Linux platform with the MariaDB database to facilitate efficient management, search, and display. The web pages were built using HTML5, CSS3, JavaScript, and Bootstrap3 (a free and open-source CSS framework directed at responsive, mobile-first front-end web development; https://getbootstrap.com/docs/3.3/). The Bootstrap-table (an extended Bootstrap table with radio, checkbox, sort, pagination, extensions, and other added features; https://bootstrap-table.com/) and jQuery (a JavaScript library designed to simplify HTML DOM tree traversal and manipulation; http://jquery.com, version 3.4.1) were used to display the query results dynamically. Presentation of the diagram was made by Echart (a free, powerful charting and visualization library offering a way of easily adding intuitive, interactive, and highly customizable charts; https://echarts.apache.org/zh/index.html).

## Utility and discussion

### Browsing RPGD

Users can browse all data in RPGD easily on the “Browse” page, including genome statistics, gene models, gene function annotations, SSRs, genome syntenic blocks, gene expression profiles, gene families and transcription factor information from *R. delavayi*, *R. williamsianum* and *R. simsii,* respectively. The information described above is presented in tabular form on the web page using a Bootstrap-table plug. Additionally, a detailed information page for a specific gene can be accessed by clicking the gene ID hyperlink. Information about each gene is displayed on a detailed page, including the gene summary, exons, gene structure (in JBrowse), GO, family, expression, homology, and sequence information.

### Searching RPGD

A series of search tools are presented on the navigation menu “Search”, such as “Gene”, “Genome”, “Gene Ontology”, “Gene Family”, “Gene Expression”, “Transcription Factor”, “Chloroplast Genome” and “SSR” to help users more easily find data of interest to them. (i). “Search Gene”: RPGD provides four different ways to search genes including gene ID, AHRD descriptions, InterPro, GO accession, and GO term. The response is a dynamic table that contains all genes associated with the entered search terms, and the list of those genes can be downloaded as a TXT file for further analysis. Additionally, the details of the genes can be viewed by clicking the gene ID hyperlink. (ii). “Search Genome”: users can use scaffold/chromosome ID to search the scaffold/chromosome information. The results are divided into a list, a table, and a chromosome viewer. The list shows basic information about the chromosome, including the species, chromosome ID, and the length of the chromosome. The table displays information about all genes on the chromosome. The chromosome viewer is embedded in JBrowse to display the chromosome profile. (iii). “Search GO”: users can use gene ID, GO accession, and GO term to query GO information of a gene. The responses are a set of genes annotated with the queried functions. Similarly, users can download the list of genes and click the gene ID hyperlink to review gene details. (iv). “Search Family”: users can find genes with gene family names specified by the user. A list of genes related to this gene family are generated as the response. Users can also download the list of genes and click the gene ID hyperlink to view gene details. (v). “Search Gene Expression”: users can input gene ID of interest to search their expression patterns based on currently provided transcriptomics results. The output is a line chart that shows graphically the expression level and can be downloaded locally for further analysis. (vi). “Search Transcription Factor”: users can search for transcription factor genes by clicking transcription factor names. The responses are a list of genes annotated as transcription factors. Users can also download the list of genes and click the gene ID hyperlink to view gene details. (vii). “Search Chloroplast Genome”: users can use the gene or product name to find the information from chloroplast genes. The response is a list of detailed information about the entered keywords. In addition, the list returned contains a number of hyperlinks which allow user to view the details about that chloroplast gene at NCBI. (viii). “Search SSR”: RPGD provides SSR location, SSR type (monomer to hexamer) and SSR motif to query the SSR detailed information, including SSR ID, type, motif, size, and location. Users can click the SSR ID hyperlink to view SSR primer information. Examples are displayed below each search field that can be clicked to autofill the search keywords on every search page.

### BLAST

**Fig. 2:**
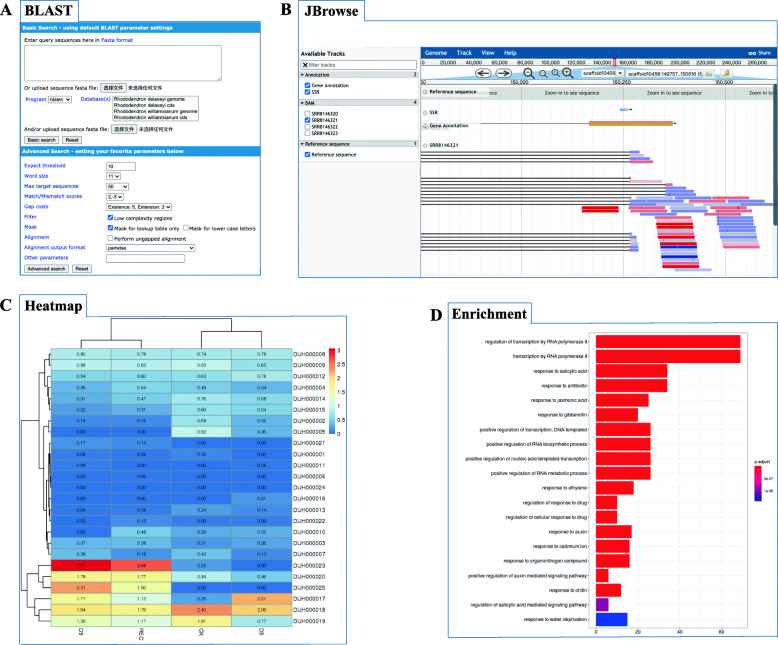
Screenshots of online tools page. **a** Online BLAST. **b** JBrowse for visualizing genome and other tracks. **c** Expression Heatmap showing expression patterns. **d** Enrichment Analysis

BLAST is a sequence similarity searching program frequently used for bioinformatics queries [46]. ViroBLAST [48], a useful and user-friendly tool for online data analysis, was integrated into RPGD (Fig. 2a). Users can input their sequence of interest or upload their sequence files to perform BLASTN, BLASTP, BLASTX, tBLASTN, and tBLASTX against a whole genome, CDS, or peptide library.

### JBrowse

A key mission of RPGD is to help users browse genomic data in detail. Therefore, JBrowse [49], a fast, scalable, and widely used genome browser built completely with JavaScript and HTML5, was embedded in RPGD to visualize genomic information (Fig. 2b). In RPGD, JBrowse hosts different tracks, including genome sequence, gene models, SSRs, and transcriptome-aligned BAM files of *R. delavayi*, *R. williamsianum*, and *R. simsii*, respectively. In addition, we will integrate other data styles, such as single-nucleotide polymorphisms (SNPs), as they become available.

### Flanking sequence finder

The flanking sequences of genes often contain a wealth of information including regulatory elements and promoters. To aid in research of flanking sequences, we utilized gene annotations and genome data to develop a useful tool - “Flanking Sequence Finder”. Researchers can find and download flanking sequences by inputting gene ID and specifying the length of the desired flanking sequences.

### Genome syntenic browser

**Fig. 3:**
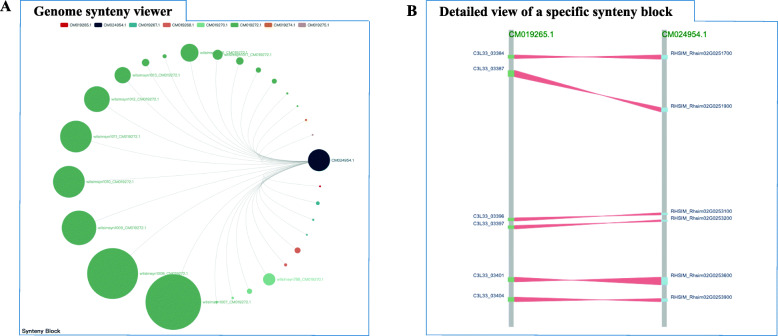
Genome synteny viewer. **a** Syntenic blocks displayed in a circus plot. The darkslategray circle represents the query chromosome. Besides, the same color represents the same chromosome, and different circles of the same color represent different syntenic blocks located on the same chromosome. Additionally, the lines between darkslategray and other colors represent syntenic blocks identified between the two genomes. By the way, all blocks of this chromosome can be made to disappear from the image by clicking on the color that represents that chromosome. **b** Detailed view of a specific synteny block. The two gray lines represent the chromosomes of different species, and the red areas represent homologous gene pairs

To view genome syntenic blocks and homologous gene pairs between the three *Rhododendron* genomes, we constructed the “Genome Syntenic Browser” module using AJAX, JavaScript and Echart. Users can browse the genome syntenic blocks or search for a specific block they want to query. Users can retrieve syntenic blocks by selecting a chromosome and subject genome together. This module returns an image to displaying all syntenic blocks for every paired query and subject genome (Fig. 3a) and a full list of the syntenic blocks. For each syntenic block, users can jump to a new page by clicking on the block ID hyperlink which contains an image to display the homologous gene pairs (Fig. 3b). The full list of genes is also provided with links to the “data hub” interface to detail the gene information for each gene (Fig. 1).

### Orthologous groups

A common task in routine bioinformatics analysis is the identification of homologous genes. Users can input gene IDs to find orthologous groups in *R. delavayi*, *R. williamsianum, R. simsii*, as well as *A. chinensis*, *C. sinensis*, and *A. thaliana*. The details of the homologous genes are be presented in a table, which also provides links to “data hub” page for each gene (Fig. 1).

### Expression heatmap

RPGD not only stores gene expression profiles derived from RNA-Seq datasets but also provides an “Expression Heatmap” module (Fig. 2c). “Expression Heatmap” can be used to retrieve the gene expression patterns of a group of genes from different samples. The output is a heatmap that graphically shows expression levels and can be downloaded locally for further analysis.

### GO and KEGG enrichment analysis

Functional enrichment analysis is a powerful method for mining gene data, providing further insight into what biological processes these genes may be involved in. To help users to capture biological information of genes, we construct the GO and KEGG enrichment analysis tools base on the functional annotation mentioned above and clusterProfiler R package [50]. Users can input a list of interested genes to perform the enrichment analysis (Fig. 2d). The results returned the significantly enriched functional categories.

### Download and batch download

All the data in RPGD were available for users to download, including genome assembly (FASTA), gene prediction (GFF3), gene function annotation (TXT), complete chloroplast genome (FASTA), gene family data (CSV), orthologous groups data (CSV), simple sequence repeat data (TXT), gene expression data (CSV), and other related data can also be downloaded in this module. “Batch Download” is provided for users to export custom datasets or bulk download datasets from RPGD. Users can download multiple types of sequences (gene, CDS, PEP, flanking sequence and gene expression profile) by inputting a list of genes.

## Conclusions

RPGD is dedicated to providing a comprehensive database of *Rhododendron* omics data. The current implementation of RPGD integrates important data including genome sequence assemblies, gene expression profiles, functional annotations, gene families, transcription factors, homologous genes, simple sequence repeats, and chloroplast genome assemblies. It also provides a series of tools for online data analysis and visualization. The integration of these data and tools makes RPGD a valuable database. We intend to continue updating the datasets when new data are released. For instance, our team will release a novel *Rhododendron* genome (*R. irroratum*) and its phenotypic datasets, including breeds, genotypes, and phenotypes in the near future. Additionally, we will continue to develop and integrate tools for functional, evolutionary, and network analysis. We hope that researchers will take advantage of these resources and also provide comments and suggestions for improving RPGD. Believe that RPGD will be in indispensable hub for *Rhododendron* studies.

## Data Availability

RPGD is freely available at http://bioinfor.kib.ac.cn/RPGD/.

## Abbreviations

RPGD: *Rhododendron* Plant Genome Database
GFF3: General feature format
CDS: Coding sequence
PEP: Protein sequence
GO: Gene ontology
TF: Transcription factor
TR: Transcriptional regulator
SSR: Simple sequence repeat
SRA: Sequence Read Archive
FPKM: Fragments per kilobase of transcript per million mapped fragments
SNP: Single-nucleotide polymorphisms

## References

[1] Yan LJ, Liu J, Möller M, Zhang L, Zhang XM, Li DZ, Gao LM (2015). DNA barcoding of *Rhododendron* (Ericaceae), the largest Chinese plant genus in biodiversity hotspots of the Himalaya-Hengduan Mountains. Mol Ecol Resour.

[2] Chamberlain D, Hyam R, Argent G, Fairweather G, Walter KS (1996). The genus *Rhododendron*: its classification and synonymy.

[3] Tian XL, Chang YH, Neilsen J, Wang SH, Ma YP (2019). A new species of *Rhododendron* (Ericaceae) from northeastern Yunnan. China Phytotaxa.

[4] Fang MY, Fang RZ, He MY, Hu LZ, Yang HB, Qin HN, Min TL, Chamberlain D, Stevens P, Wallace G, Anderberg A (2005). Flora of China. Volume 14: Apiaceae through Ericaceae.

[5] Ma YP, Wu ZK, Xue RJ, Tian XL, Gao LM, Sun WB (2013). A new species of *Rhododendron* (Ericaceae) from the Gaoligong Mountains, Yunnan, China, supported by morphological and DNA barcoding data. Phytotaxa..

[6] De RJ, De KE, Calsyn E, Eeckhaut T, Van HJ, Kobayashi N, Van HJ (2018). Azalea. Ornamental Crops.

[7] Popescu R, Kopp B (2013). The genus *Rhododendron*: an ethnopharmacological and toxicological review. J Ethnopharmacol.

[8] Yonghui Z, Weibing J, Mangling W (2007). Meanings of *Rhododendron* and ways used in gardens. Chin Agric Sci Bull.

[9] Kron KA, Gawen LM, Chase MW, et al. Evidence for introgression in azaleas (*Rhododendron*; Ericaceae): Chloroplast DNA and morphological variation in a hybrid swarm on Stone Mountain, Georgia. Am J Bot. 1993;80(9):1095–9. 10.1002/j.1537-2197.1993.tb15335.x.

[10] Leslie A (2004). The international *Rhododendron* register and checklist.

[11] Cox PA (1979). The larger species of rhododendron.

[12] Perkins S (2015). More weighings: exploring the ploidy of hybrid elepidote. rhododendrons. Azalean.

[13] Qiang Y, Zhou B, Gao K (2011). Chemical constituents of plants from the genus *Rhododendron*. Chem Biodivers.

[14] Zhu YX, Zhang ZX, Yan HM, Lu D, Zhang HP, Li L, Liu YB, Li Y (2018). Antinociceptive diterpenoids from the leaves and twigs of *Rhododendron decorum*. J Nat Prod.

[15] Zhou J, Liu T, Zhang H, Zheng G, Qiu Y, Deng M, Zhang C, Yao G (2018). Anti-inflammatory grayanane diterpenoids from the leaves of *Rhododendron molle*. J Nat Prod.

[16] Zhu H, Li C, Gao C (2020). Applications of CRISPR–Cas in agriculture and plant biotechnology. Nat Rev Mol Cell Biol.

[17] Zhang L, Xu PW, Cai YF, Ma LL, Li SF, Li SF, Xie WJ, Song J, Peng LC, Yan HJ (2017). The draft genome assembly of *Rhododendron delavayi* Franch. var*. delavayi*. GigaScience.

[18] Soza VL, Lindsley D, Waalkes A, Ramage E, Patwardhan RP, Burton JN, Adey A, Kumar A, Qiu RL, Shendure J, Hall B (2019). The *Rhododendron* genome and chromosomal organization provide insight into shared whole-genome duplications across the heath family (Ericaceae). Genome Biol Evol.

[19] Yang FS, Nie S, Liu H, Shi TL, Tian XC, Zhou SS, Bao YT, Jia KH, Guo JF, Zhao W (2020). Chromosome-level genome assembly of a parent species of widely cultivated azaleas. Nat Commun.

[20] Choudhary S, Thakur S, Jaitak V, Bhardwaj P (2019). Gene and metabolite profiling reveals flowering and survival strategies in Himalayan *Rhododendron arboreum*. Gene..

[21] Xing W, Liao J, Cai M, Xia Q, Liu Y, Zeng W, Jin X (2017). De novo assembly of transcriptome from *Rhododendron latoucheae* Franch. using Illumina sequencing and development of new EST-SSR markers for genetic diversity analysis in *Rhododendron*. Tree Genet Genomes.

[22] Choudhary S, Thakur S, Najar RA, Majeed A, Singh A, Bhardwaj P (2018). Transcriptome characterization and screening of molecular markers in ecologically important Himalayan species (*Rhododendron arboreum*). Genome..

[23] Cai YF, Wang JH, Zhang L, Song J, Peng LC, Zhang SB (2019). Physiological and transcriptomic analysis highlight key metabolic pathways in relation to drought tolerance in *Rhododendron delavayi*. Physiol Mol Biol Plants.

[24] Jia X, Tang L, Mei X, Liu H, Luo H, Deng Y, Su J (2020). Single-molecule long-read sequencing of the full-length transcriptome of *Rhododendron lapponicum* L. Sci Rep.

[25] Sayers EW, Beck J, Brister JR, Bolton EE, Canese K, Comeau DC, Funk K, Ketter A, Kim S, Kimchi A, Kitts PA, Kuznetsov A, Lathrop S, Lu Z, McGarvey K, Madden TL, Murphy TD, O’Leary N, Phan L, Schneider VA, Thibaud-Nissen F, Trawick BW, Pruitt KD, Ostell J (2020). Database resources of the National Center for Biotechnology Information. Nucleic Acids Res.

[26] Sneddon TP, Li P, Edmunds SC. GigaDB: announcing the GigaScience database. GigaScience. 2012. 10.1186/2047-217X-1-11. 10.1186/2047-217X-1-11 PMC3626507 23587345

[27] Leinonen R, Sugawara H, Shumway M, on behalf of the International Nucleotide Sequence Database Collaboration (2011). The sequence read archive. Nucleic Acids Res.

[28] Bolger AM, Lohse M, Usadel B (2014). Trimmomatic: a flexible trimmer for Illumina sequence data. Bioinformatics..

[29] Kim D, Pertea G, Trapnell C, Pimentel H, Kelley R, Salzberg SL (2013). TopHat2: accurate alignment of transcriptomes in the presence of insertions, deletions and gene fusions. Genome Biol.

[30] Trapnell C, Williams BA, Pertea G, Mortazavi A, Kwan G, van Baren MJ, Salzberg SL, Wold BJ, Pachter L (2010). Transcript assembly and quantification by RNA-Seq reveals unannotated transcripts and isoform switching during cell differentiation. Nat Biotechnol.

[31] Huerta-Cepas J, Forslund K, Coelho LP, Szklarczyk D, Jensen LJ, von Mering C, Bork P (2017). Fast genome-wide functional annotation through orthology assignment by eggNOG-mapper. Mol Biol Evol.

[32] Huerta-Cepas J, Szklarczyk D, Heller D, Hernandez-Plaza A, Forslund SK, Cook H, Mende DR, Letunic I, Rattei T, Jensen LJ (2019). eggNOG 5.0: a hierarchical, functionally and phylogenetically annotated orthology resource based on 5090 organisms and 2502 viruses. Nucleic Acids Res.

[33] Mitchell AL, Attwood TK, Babbitt PC, Blum M, Bork P, Bridge A, Brown SD, Chang HY, El-Gebali S, Fraser MI (2019). InterPro in 2019: improving coverage, classification and access to protein sequence annotations. Nucleic Acids Res.

[34] Buchfink B, Xie C, Huson DH (2015). Fast and sensitive protein alignment using DIAMOND. Nat Methods.

[35] Zheng Y, Jiao C, Sun HH, Rosli Hernan G, Pombo Marina A, Zhang P, Banf M, Dai XB, Martin Gregory B, Giovannoni James J (2016). iTAK: a program for genome-wide prediction and classification of plant transcription factors, transcriptional regulators, and protein kinases. Mol Plant.

[36] Emms DM, Kelly S (2015). OrthoFinder: solving fundamental biases in whole genome comparisons dramatically improves orthogroup inference accuracy. Genome Biol.

[37] Emms DM, Kelly S (2019). OrthoFinder: phylogenetic orthology inference for comparative genomics. Genome Biol.

[38] Huang S, Ding J, Deng D, Tang W, Sun H, Liu D, Zhang L, Niu X, Zhang X, Meng M, Yu J, Liu J, Han Y, Shi W, Zhang D, Cao S, Wei Z, Cui Y, Xia Y, Zeng H, Bao K, Lin L, Min Y, Zhang H, Miao M, Tang X, Zhu Y, Sui Y, Li G, Sun H, Yue J, Sun J, Liu F, Zhou L, Lei L, Zheng X, Liu M, Huang L, Song J, Xu C, Li J, Ye K, Zhong S, Lu BR, He G, Xiao F, Wang HL, Zheng H, Fei Z, Liu Y (2013). Draft genome of the kiwifruit Actinidia chinensis. Nat Commun.

[39] Xia EH, Li FD, Tong W, Li PH, Wu Q, Zhao HJ, Ge RH, Li RP, Li YY, Zhang ZZ, Wei CL, Wan XC (2019). Tea plant information archive: a comprehensive genomics and bioinformatics platform for tea plant. Plant Biotechnol J.

[40] Lamesch P, Berardini TZ, Li DH, Swarbreck D, Wilks C, Sasidharan R, Muller R, Dreher K, Alexander DL, Garcia-Hernandez M, Karthikeyan AS, Lee CH, Nelson WD, Ploetz L, Singh S, Wensel A, Huala E (2012). The Arabidopsis information resource (TAIR): improved gene annotation and new tools. Nucleic Acids Res.

[41] Beier S, Thiel T, Munch T, Scholz U, Mascher M (2017). MISA-web: a web server for microsatellite prediction. Bioinformatics..

[42] Untergasser A, Cutcutache I, Koressaar T, Ye J, Faircloth BC, Remm M, Rozen SG (2012). Primer3-new capabilities and interfaces. Nucleic Acids Res.

[43] Liu J, Chen T, Zhang YB, Li YK, Gong JY, Yi Y (2020). The complete chloroplast genome of *Rhododendron delavayi* (Ericaceae). Mitochondrial DNA Part B-Resour.

[44] Li HE, Guo QQ, Li Q, Yang L. Long-reads reveal that *Rhododendron delavayi* plastid genome contains extensive repeat sequences, and recombination exists among plastid genomes of photosynthetic Ericaceae. Peerj. 2020. 10.7717/peerj.9048. 10.7717/peerj.9048 PMC7183307 32351791

[45] Shen JS, Li XQ, Zhu XT, Huang XL, Jin SH (2019). Complete chloroplast genome of *Rhododendron pulchrum*, an ornamental medicinal and food tree. Mitochondrial DNA Part B-Resour.

[46] Altschul SF, Gish W, Miller W, Myers EW, Lipman DJ (1990). Basic local alignment search tool. J Mol Biol.

[47] Wang YP, Tang HB, DeBarry JD, Tan X, Li JP, Wang XY, Lee TH, Jin HZ, Marler B, Guo H (2012). MCScanX: a toolkit for detection and evolutionary analysis of gene synteny and collinearity. Nucleic Acids Res.

[48] Deng W, Nickle DC, Learn GH, Maust B, Mullins JI (2007). ViroBLAST: a stand-alone BLAST web server for flexible queries of multiple databases and user's datasets. Bioinformatics..

[49] Buels R, Yao E, Diesh CM, Hayes RD, Munoz-Torres M, Helt G, Goodstein DM, Elsik CG, Lewis SE, Stein L, Holmes IH (2016). JBrowse: a dynamic web platform for genome visualization and analysis. Genome Biol.

[50] Yu G, Wang LG, Han Y, He QY (2012). clusterProfiler: an R package for comparing biological themes among gene clusters. Omics..

